# Post-transplant oncologic outcomes after locoregional downstaging before living donor liver transplantation for hepatocellular carcinoma beyond Milan criteria: a systematic review and meta-analysis of selected cohorts

**DOI:** 10.3389/ti.2026.17284

**Published:** 2026-10-20

**Authors:** Younggoun Jo, Yunchul Park, Ho-Kyun Lee, Sola Lee, Hong-Sung Jung, Soo Jin Na Choi, Hyo-Sin Kim

**Affiliations:** Department of Surgery, Chonnam National University Medical School and Hospital, Gwangju, Republic of Korea

**Keywords:** downstaging, hepatocellular carcinoma, living donor liver transplantation, locoregional therapy, meta-analysis

## Abstract

Downstaging of hepatocellular carcinoma (HCC) before liver transplantation is established mainly in the deceased donor setting, and outcomes before living donor liver transplantation (LDLT) remain unclear. We searched PubMed and Embase through April 2026 for studies reporting post-transplant outcomes in patients with HCC beyond Milan criteria who were downstaged before LDLT. A pre-specified primary analysis covered locoregional therapy targeting Milan or UCSF criteria. We pooled proportions using random-effects models and reconstructed patient-level survival from Kaplan-Meier curves. Seventeen studies with 511 downstaged LDLT recipients were included. In the primary cohort, recurrence was 22.1% (95% CI 9.7–42.6) and 5-year overall survival 75.8% (61.8–85.8). Across all studies, recurrence was 25.0% (17.4–34.5), 5-year overall survival 75.0% (63.1–84.1), and downstaging success 47.5% (36.1–59.1); reconstructed 5-year overall and recurrence-free survival were 75.7% and 75.6%. These estimates describe patients who achieved downstaging—fewer than half of those who attempted it—and then reached transplant. They do not measure the benefit of downstaging itself. The evidence remains retrospective and mostly Asian, with wide variation between studies. Prospective intention-to-treat studies are needed.

## Graphical Abstract

**Figure:**
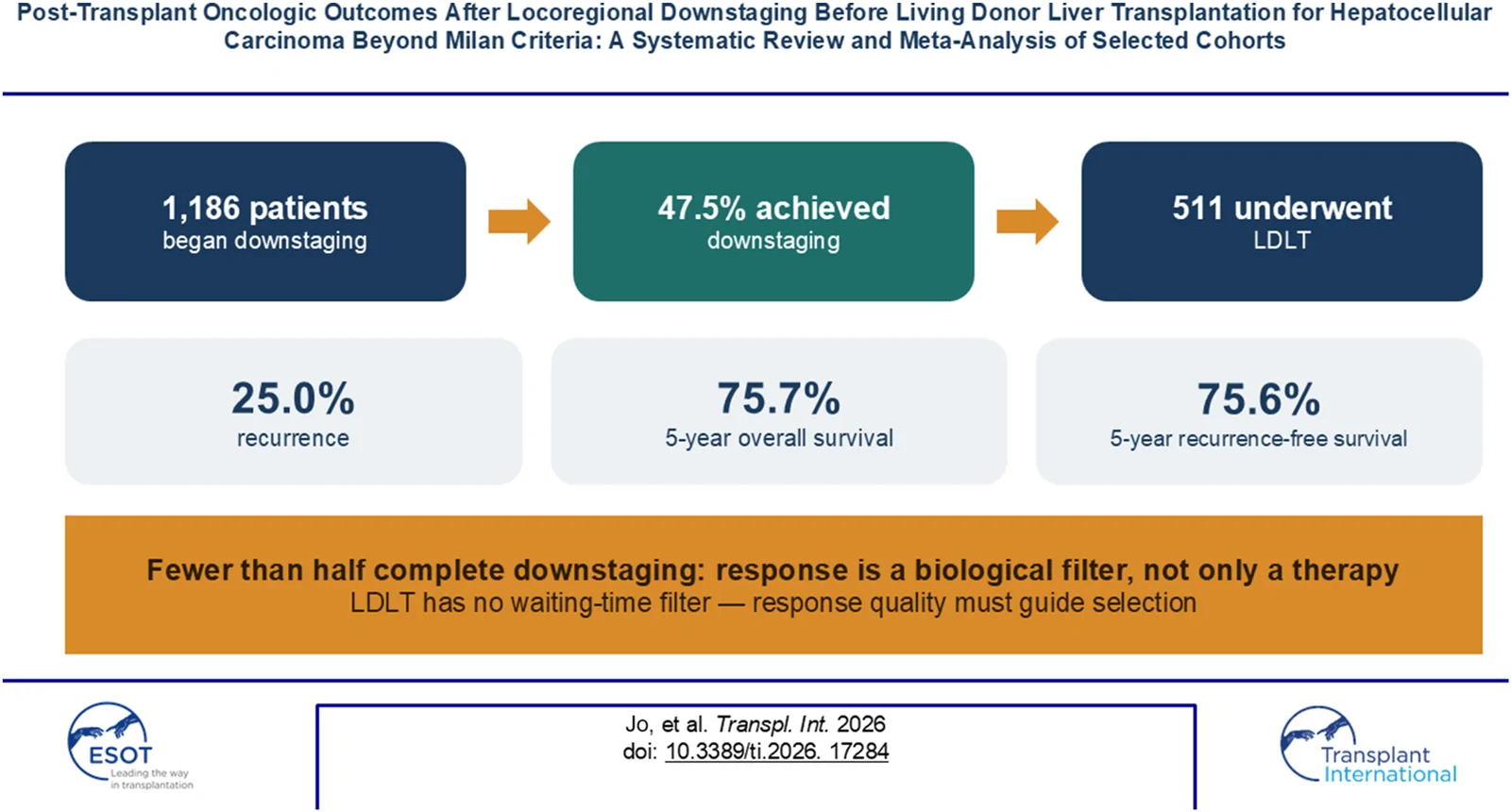

## Introduction

Hepatocellular carcinoma (HCC) is the sixth most common cancer and the third leading cause of cancer-related death worldwide, with approximately 865,000 new cases and 758,000 deaths annually [1]. Liver transplantation (LT) removes both the tumor and the diseased liver. Since Mazzaferro et al. in 1996, the Milan criteria—a single tumor ≤5 cm or up to three tumors each ≤3 cm, without macrovascular invasion or extrahepatic spread—have been the benchmark for selection, with 5-year survival above 70% [2].

Strict adherence to the Milan criteria excludes many patients who might benefit from transplantation. Expanded criteria followed, such as the University of California San Francisco (UCSF) criteria, the Metroticket 2.0 model, and Asian criteria including Asan, Kyoto (5-5-500), and Seoul National University Hospital (SNUH) [3–5]. In parallel, downstaging—reducing tumor burden with locoregional therapy (LRT) to meet transplant criteria—has emerged as a way to expand candidacy [6, 7].

The evidence for downstaging comes predominantly from deceased donor liver transplantation (DDLT). The UCSF downstaging protocol, the 2017 United Network for Organ Sharing/Organ Procurement and Transplantation Network (UNOS/OPTN) national policy, and the XXL randomized trial all showed that successfully downstaged patients reach post-transplant survival close to that of patients within Milan criteria from the start [7–9], and meta-analyses of these mostly DDLT data report 5-year survival of 60%–80% [10–12].

Living donor liver transplantation (LDLT) is a different paradigm. It accounts for most liver transplants in Asia, where deceased donation remains low [13, 14]. LDLT does not depend on a shared allocation system, so centers have more freedom in selection and timing; many Asian centers accept patients beyond conventional limits based on alpha-fetoprotein (AFP), des-gamma-carboxy prothrombin (DCP), and response to pretransplant therapy [15, 16]. A healthy donor also assumes surgical risk, which makes a clear account of expected oncological outcome an ethical requirement.

We therefore reviewed and pooled post-transplant oncological outcomes after locoregional downstaging before LDLT for HCC beyond Milan criteria, with a pre-specified primary analysis restricted to studies targeting standard radiologic criteria (Milan or UCSF). We asked whether the oncological signal that justifies downstaging in DDLT survives the greater selection freedom of LDLT, and which pooled outcome is most comparable across this clinically diverse literature.

## Methods

### Study registration and reporting

This review followed the Preferred Reporting Items for Systematic Reviews and Meta-Analyses (PRISMA) 2020 guidelines [17] and was prospectively registered in PROSPERO (CRD420261368824). This study was approved by the Institutional Review Board of Chonnam National University Hospital (CNUH-EXP-2026-138). As this was a systematic review and meta-analysis of published studies, informed consent was waived.

### Search strategy

We searched PubMed (including MEDLINE) and Embase from inception through April 8, 2026, combining three concept blocks: living donor liver transplantation, hepatocellular carcinoma, and downstaging or locoregional therapy (Supplementary Table S1). We supplemented this with Google Scholar, manual review of the reference lists of included articles and relevant reviews, and forward citation tracking. No language or date restrictions were applied.

### Eligibility criteria

Studies were included if they (1) enrolled patients with HCC beyond Milan criteria at initial presentation; (2) applied downstaging therapy (locoregional, systemic, or combined) with the intent to achieve transplant eligibility; (3) transplanted these patients by LDLT; and (4) reported at least one of OS, RFS or disease-free survival (DFS), recurrence rate, or downstaging success rate. We excluded studies reporting DDLT without separable LDLT data; reviews, editorials, and conference abstracts without original data; studies of bridging therapy for patients already within Milan criteria; and studies with fewer than 4 downstaged LDLT patients. Where publications from one institution overlapped, we kept the study with the largest sample or most complete outcome data.

### Study selection and data extraction

The primary investigator screened titles and abstracts, then full texts, against predefined criteria. To reduce the error risk of single-reviewer screening, a pilot of 20 randomly selected abstracts was screened first to calibrate the criteria, and ambiguous cases were resolved by re-reading the source publications. Data were extracted on a standardized form, and every numerical value was cross-checked against the source publication a second time. Extracted variables included author, year, country, design, study period, patient numbers, tumor burden, downstaging modality, selection criteria, success rate, OS and RFS/DFS at 1, 3, and 5 years, recurrence, follow-up, and AFP.

### Quality assessment

Methodological quality was assessed with the Newcastle-Ottawa Scale (NOS) for cohort studies (selection 4 points, comparability 2, outcome 3; maximum 9). Scores of 7–9 were classified as high, 4–6 as moderate, and 0–3 as low quality.

### Statistical analysis

Pooled proportions were calculated by the inverse variance method with logit transformation [18] under random-effects (DerSimonian-Laird) models. For OS and RFS, event numbers were estimated from reported survival proportions and sample sizes; a continuity correction of 0.5 was applied to zero-event studies. Heterogeneity was quantified with the I^2^ statistic and Cochran’s Q test, with I^2^ of <25%, 25%–50%, 50%–75%, and >75% denoting low, moderate, substantial, and considerable heterogeneity.

Subgroup analysis was performed by presence of portal vein tumor thrombosis (PVTT) or macrovascular invasion (MVI). Leave-one-out sensitivity analysis assessed the influence of individual studies. Publication bias was assessed with funnel plots and Egger’s linear regression test. Analyses used R version 4.3 (R Foundation for Statistical Computing) with the “meta” (version 8.3) and “metafor” (version 4.8) packages; a two-sided p-value <0.05 was considered significant.

Because pooled proportions ignore censoring and uneven follow-up, we also reconstructed individual patient data (IPD) from published Kaplan–Meier curves by the method of Guyot et al. [19] for every study with a survival curve for the downstaged cohort. Digitized curve coordinates were combined with the reported numbers at risk, or with reported survival proportions at fixed time points where a risk table was missing. Reconstructed data were pooled in a one-stage analysis, and overall and recurrence-free survival were estimated by the Kaplan–Meier method with Greenwood confidence intervals. Reconstruction reproduced each study’s reported survival rates to within four percentage points.

Two denominators are involved. Survival and recurrence were calculated among patients who completed both successful downstaging and LDLT, whereas the downstaging success rate was calculated among every patient who attempted downstaging. Survival estimates therefore describe a selected cohort of successfully treated patients.

### Primary and secondary analyses

A pre-specified primary analysis was restricted to LRT-based downstaging protocols targeting standard radiologic criteria (Milan or UCSF). Studies focused on PVTT or macrovascular invasion using radiation-based protocols (Soin 2020, Han 2016, Jeong 2017), immune checkpoint inhibitor (ICI)-based regimens (Kulkarni 2024, Cheng 2025), and expanded biology-based criteria without standard radiologic endpoints (Llovet 2018, Ogawa 2019, Bhatti 2022) were analyzed as secondary or exploratory analyses. This stratification reduces clinically driven heterogeneity.

## Results

### Study selection

The search identified 109 records (85 from PubMed/MEDLINE, 18 from Embase, and 6 from reference lists and citation tracking). After removal of 7 duplicates, 102 records were screened by title and abstract and 56 were excluded. Of 46 full-text articles, 29 were excluded: no or incomplete downstaging data (n = 8), review/editorial/letter (n = 6), LDLT data not separable from DDLT (n = 3), fewer than 4 patients (n = 4), within Milan criteria or bridging therapy only (n = 4), and other (n = 4). Seventeen studies were included in the quantitative synthesis (Figure 1).

**FIGURE 1:**
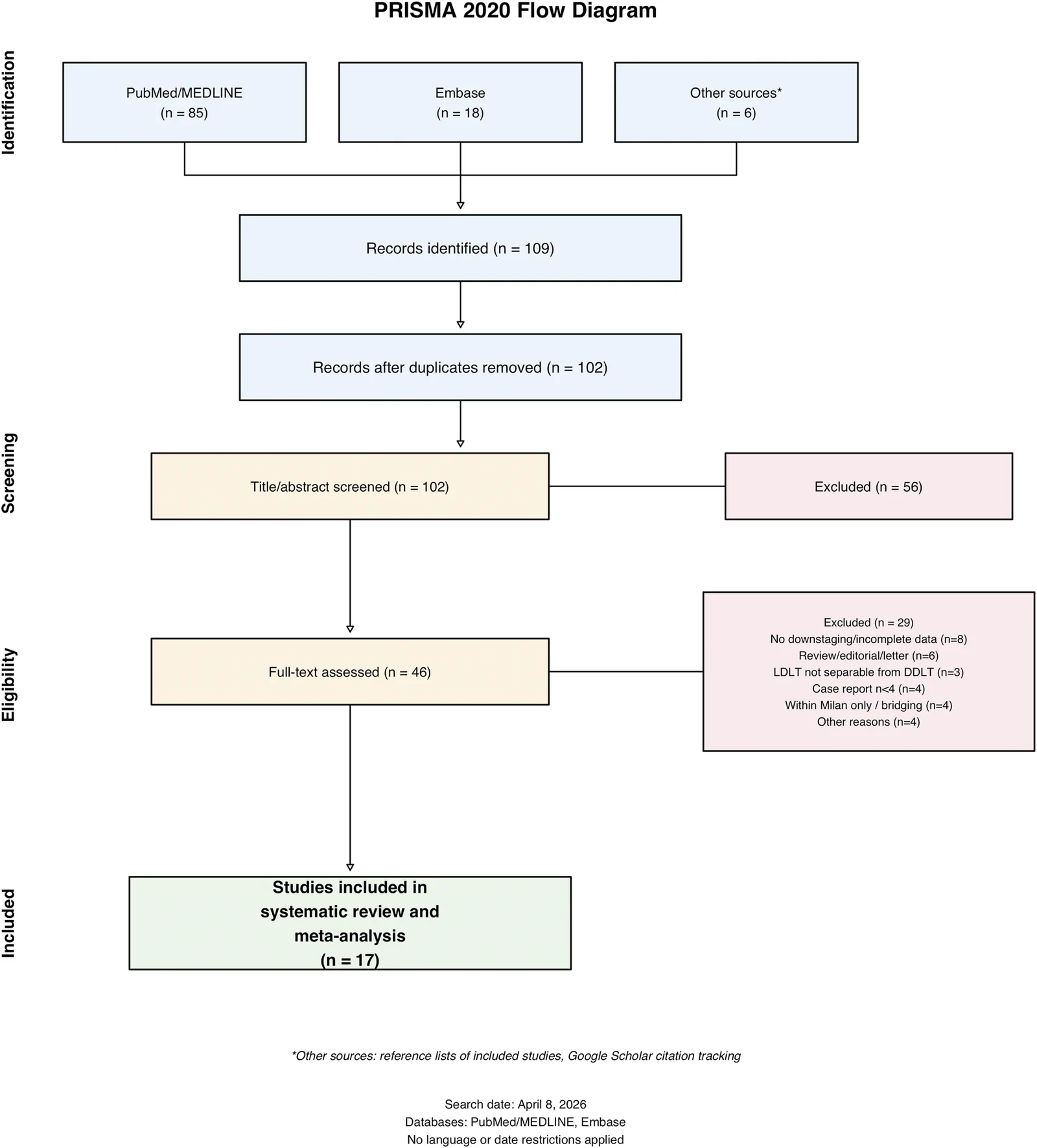
PRISMA 2020 flow diagram of study selection. A total of 109 records were identified, and 17 studies were ultimately included in the quantitative synthesis.

### Study characteristics

Table 1 summarizes the 17 included studies, published between 2010 and 2025, from South Korea (n = 6), Taiwan (n = 4), Japan (n = 2), India (n = 2), China (n = 1), Spain (n = 1), and Pakistan (n = 1). Fifteen were retrospective and two prospective. Downstaged LDLT recipients numbered 4 to 144 per study. The most common modalities were transarterial chemoembolization (TACE) variants—conventional TACE, drug-eluting bead TACE (DEB-TACE), and transarterial chemo-lipiodolization (TACL)—alone or combined with radiofrequency ablation (RFA), radiation therapy, or hepatic resection. Three studies addressed tumors with PVTT using radiation-based protocols, and two recent studies used ICI-based regimens.

**TABLE 1:** Characteristics of included studies.

Study	Country	Design	Period	n	PVTT	Downstaging modality	Success rate	FU, mo
Soin 2020	India	Prosp	2006–2017	25	Yes	SBRT/TARE/TACE/RFA	63% (27/43)	33
Bhatti 2022	Pakistan	Retro	2012–2019	27	Yes	TACE/RFA/MWA	NR	28
Lei 2012	China	Retro	2003–2009	44	No	TACE/resection/RFA/EI	61% (44/72)	72
Han 2016	Korea	Retro	2011–2012	8	Yes	CCRT/HAIC	100% (8/8)	17
Jeong 2017	Korea	Retro	2007–2014	17	Yes	3D-CRT/TACE	NR	24
Inomata 2021	Japan	Retro	2000–2018	4	No	TACE/RFA/MCT	44% (4/9)	62
Na 2016	Korea	Retro	2003–2012	12	No	TACE/RFA/resection/PEI	36% (12/33)	37
Llovet 2018	Spain	Prosp	2001–2014	22	No	TACE/PEI	100% (5/5)	81
Kulkarni 2024	India	Retro	2020–2023	4	Yes	Atezo-Bev	10% (12/115)	10
Lee S 2020	Korea	Retro	2005–2015	144	No	cTACE/RFA/combination	68% (144/213)	89
Cho 2018	Korea	Retro	2002–2015	37	No	TACE	NR	44
Ogawa 2019	Japan	Retro	1999–2015	8	No	TACE/TAE/RFA/PEI/resection	38% (8/21)	90
Jang 2010	Korea	Retro	2000–2007	37	No	TACL	42% (160/386)	29
Wu 2020	Taiwan	Retro	2004–2018	28	No	TACE/ablation	21% (6/28)	NR
Yu 2012	Taiwan	Retro	2004–2010	51	No	LRT (mixed)	32% (51/161)	29
Lim 2024	Taiwan	Retro	2012–2020	33	No	DEB-TACE	58% (139/238)	NR
Cheng 2025	Taiwan	Retro	2021–2024	10	Yes	ICI	NR	16

Retro = retrospective; Prosp = prospective; PVTT, portal vein tumor thrombosis; FU, follow-up; NR, not reported; TACE, transarterial chemoembolization; RFA, radiofrequency ablation; SBRT, stereotactic body radiation therapy; TARE, transarterial radioembolization; HAIC, hepatic arterial infusion chemotherapy; DEB-TACE, drug-eluting bead TACE; ICI, immune checkpoint inhibitor; Atezo-Bev = atezolizumab-bevacizumab; LRT, locoregional therapy; n = number of downstaged LDLT recipients.

Target criteria varied: Milan in 8 studies, UCSF in 4, and institution-specific criteria (Kyoto 5-5-500, BCLC expanded, PVTT response-based) in the remainder. Nine studies met the primary-analysis definition (LRT-based protocols, Milan or UCSF radiologic endpoint, no predominant PVTT or macrovascular invasion focus): Lei 2012, Inomata 2021, Na 2016, Lee S 2020, Cho 2018, Jang 2010, Wu 2020, Yu 2012, and Lim 2024. The remaining eight (three PVTT-focused, two ICI-based, and three with expanded or institution-specific criteria; see Methods) were analyzed as secondary or exploratory. Reported follow-up ranged from 10 to 90 months (median in most studies; mean in two).

### Quality assessment

NOS scores ranged from 4 to 9, with a mean of 7.2 ± 1.7 (Table 2). Eleven studies (64.7%) were high quality (NOS ≥7) and six (35.3%) moderate quality (NOS 4–6); none was low quality. The most common limitation was the absence of a comparison group (single-arm design), seen in 7 studies.

**TABLE 2:** Risk of bias assessment (Newcastle-Ottawa Scale).

Study	Selection	Comparability	Outcome	Total	Quality
Soin 2020	3/4	0/2	3/3	6/9	Moderate
Bhatti 2022	4/4	1/2	3/3	8/9	High
Lei 2012	4/4	1/2	3/3	8/9	High
Han 2016	2/4	0/2	2/3	4/9	Moderate
Jeong 2017	3/4	0/2	3/3	6/9	Moderate
Inomata 2021	4/4	1/2	3/3	8/9	High
Na 2016	4/4	2/2	3/3	9/9	High
Llovet 2018	3/4	0/2	3/3	6/9	Moderate
Kulkarni 2024	2/4	0/2	2/3	4/9	Moderate
Lee S 2020	4/4	2/2	3/3	9/9	High
Cho 2018	4/4	1/2	3/3	8/9	High
Ogawa 2019	4/4	2/2	3/3	9/9	High
Jang 2010	4/4	1/2	3/3	8/9	High
Wu 2020	4/4	2/2	3/3	9/9	High
Yu 2012	4/4	1/2	3/3	8/9	High
Lim 2024	4/4	1/2	3/3	8/9	High
Cheng 2025	3/4	0/2	2/3	5/9	Moderate

Newcastle-Ottawa Scale for cohort studies: Selection (max 4), Comparability (max 2), Outcome (max 3).

Quality: High (7–9), Moderate (4–6), Low (0–3).

### Primary analysis

In the nine primary-analysis studies (LRT-based, Milan/UCSF endpoint, excluding PVTT-focused and ICI-based studies), the pooled recurrence rate was 22.1% (95% CI: 9.7%–42.6%; I^2^ = 81.4%; 5 studies, N = 173) (Figure 2A), the pooled 5-year OS was 75.8% (95% CI: 61.8%–85.8%; I^2^ = 81.3%; 6 studies, N = 337) (Figure 2B), and the pooled 5-year RFS was 72.8% (95% CI: 55.3%–85.2%; I^2^ = 77.0%; 4 studies, N = 221). The restricted recurrence estimate was broadly consistent with the overall pooled analysis (25.0%), but heterogeneity remained substantial for all three outcomes. Supplementary Table S2 lists the studies contributing to each pooled outcome.

**FIGURE 2:**
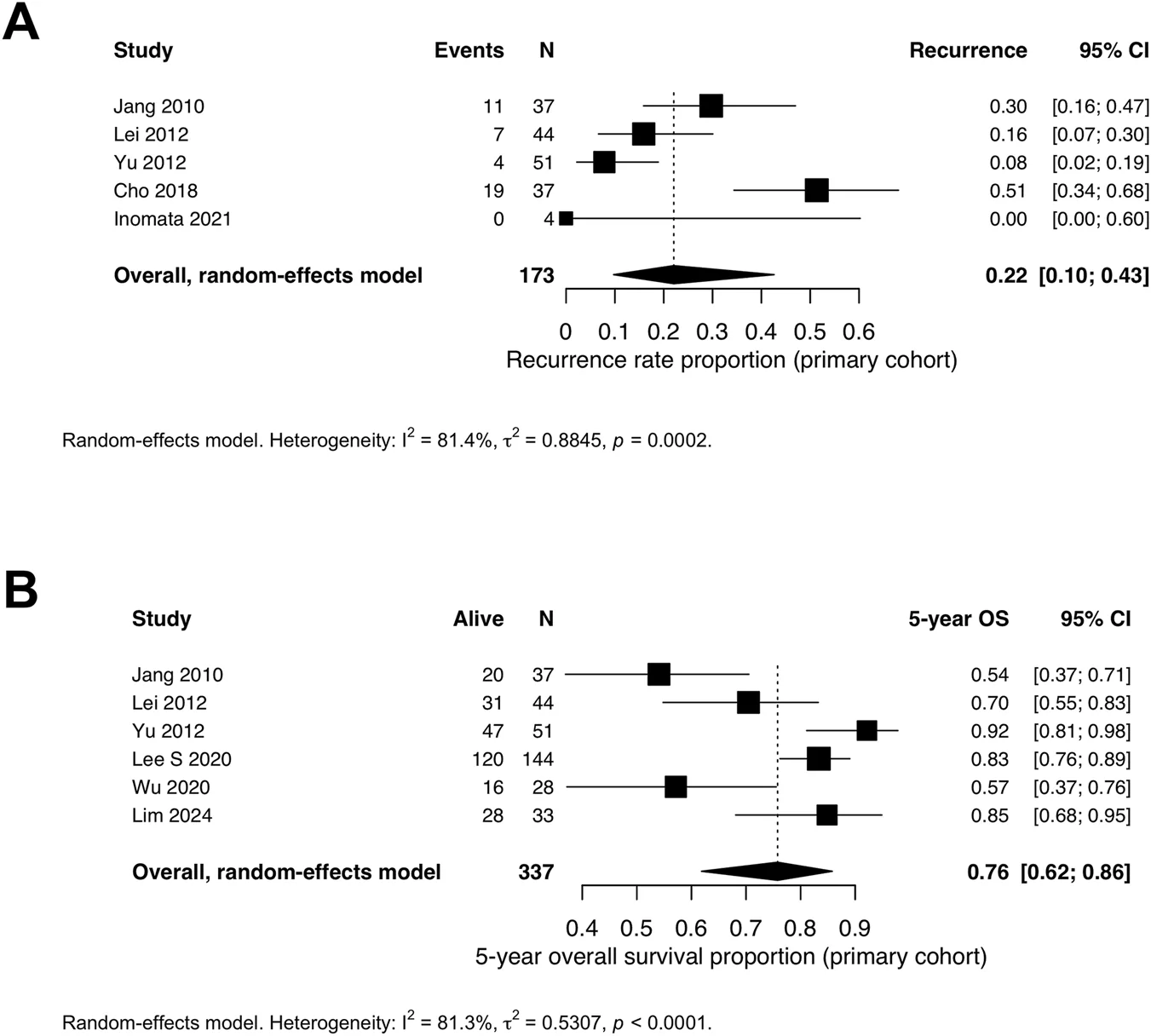
Forest plots of the pre-specified primary analysis (locoregional therapy-based downstaging targeting Milan or UCSF criteria; studies focused on portal vein tumor thrombosis or immune checkpoint inhibitor protocols excluded). **(A)** Recurrence rate: 5 studies, N = 173; pooled 22.1% (95% CI: 9.7%–42.6%; I^2^ = 81.4%). **(B)** 5-year overall survival: 6 studies, N = 337; pooled 75.8% (95% CI: 61.8%–85.8%; I^2^ = 81.3%). Diamonds indicate random-effects pooled estimates. Cho 2018 contributed the beyond-Milan subset (19/37); within-Milan bridging cases were excluded. CI, confidence interval.

### Overall analysis (all 17 studies)

Across all 17 studies, the pooled 1-year OS was 90.5% (95% CI: 85.2%–94.1%; 9 studies, 383 patients; I^2^ = 36.9%, p = 0.12) (Figure 3A), the pooled 3-year OS 77.7% (95% CI: 64.9%–86.7%; 7 studies, 293 patients; I^2^ = 70.6%, p = 0.002) (Figure 3B), and the pooled 5-year OS 75.0% (95% CI: 63.1%–84.1%; 9 studies, 392 patients; I^2^ = 77.2%, p < 0.001) (Figure 3C).

**FIGURE 3:**
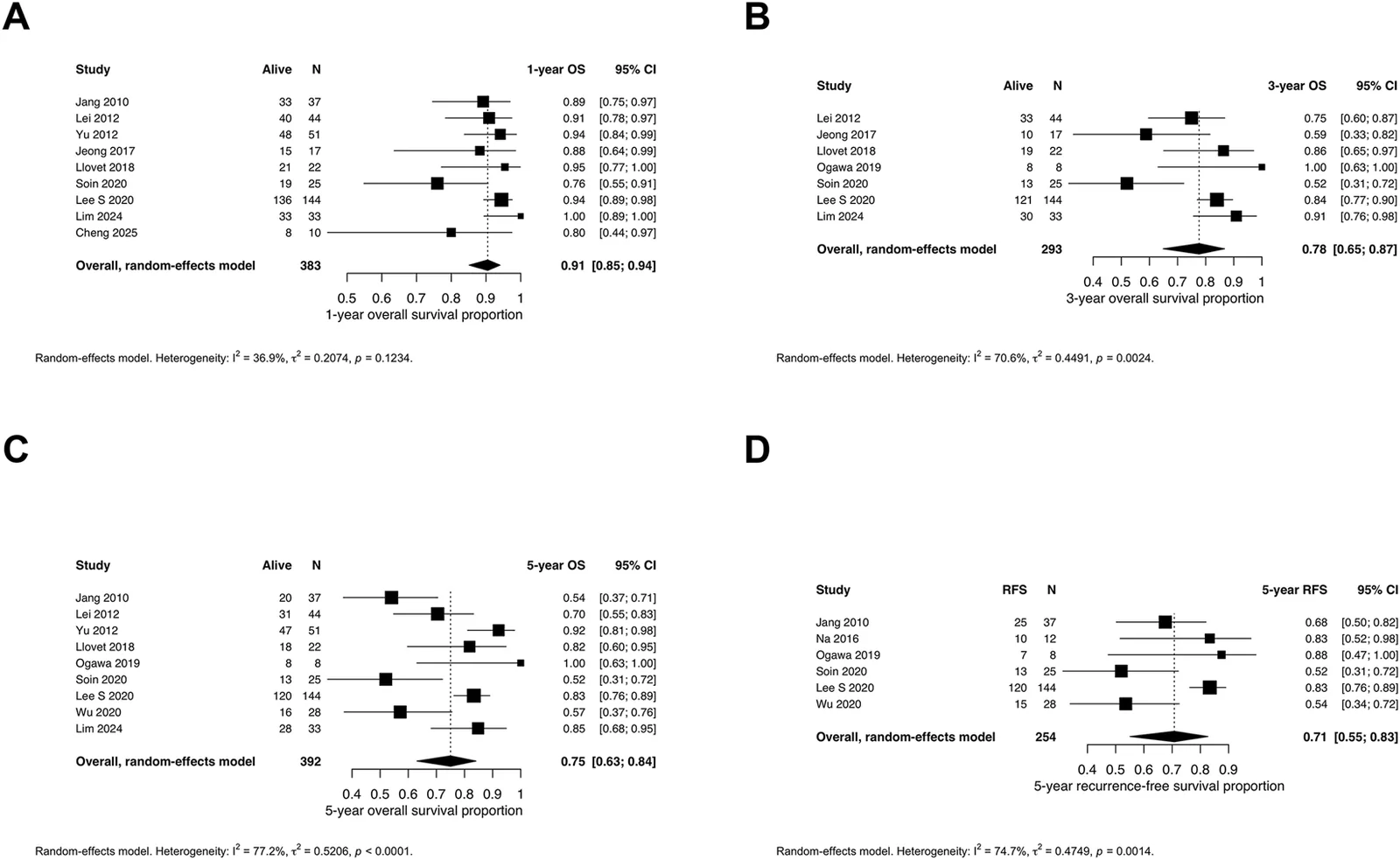
Forest plots of pooled overall survival and recurrence-free survival after downstaging and LDLT across all 17 included studies. **(A)** 1-year OS. **(B)** 3-year OS. **(C)** 5-year OS. **(D)** 5-year RFS. Diamond indicates the pooled estimate from random-effects model. CI, confidence interval.

### Recurrence-free survival

The pooled 5-year RFS was 70.8% (95% CI: 55.1%–82.7%; 6 studies, 254 patients; I^2^ = 74.7%, p = 0.001) (Figure 3D), with substantial heterogeneity reflecting differences in patient selection, downstaging protocols, and follow-up.

### Recurrence rate

The pooled recurrence rate was 25.0% (95% CI: 17.4%–34.5%; 12 studies, 286 patients; I^2^ = 55.7%, p = 0.010) (Figure 4). Individual study rates ranged from 0% (Inomata et al., Kulkarni et al., n = 4 each) to 51.4% (Cho et al., n = 37, beyond-Milan subset). Per-study outcomes are summarized in Table 3.

**FIGURE 4:**
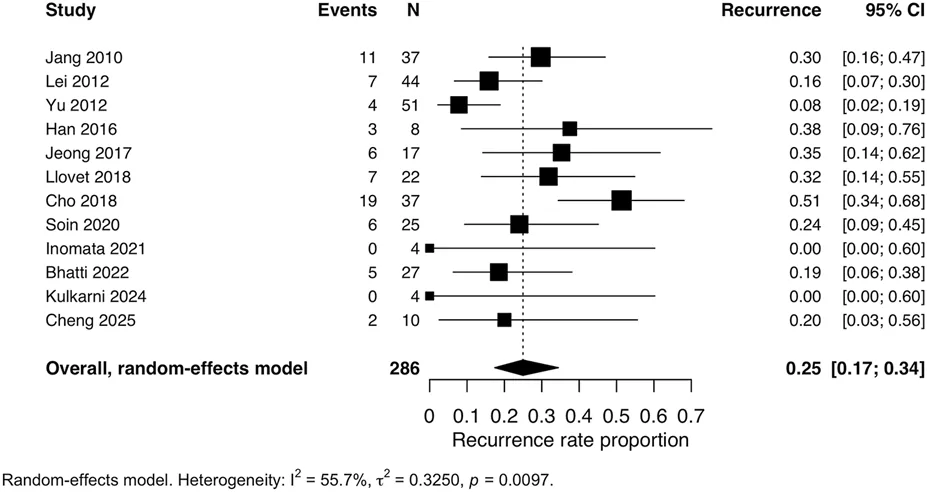
Forest plot of pooled recurrence rate after downstaging and LDLT. The pooled recurrence rate was 25.0% (95% CI: 17.4%–34.5%) with substantial heterogeneity (I^2^ = 55.7%).

**TABLE 3:** Reported oncologic outcomes of the included studies.

​	​	Overall survival (%)			Recurrence-free survival (%)			​
Study	Follow-up (mo)	1-year	3-year	5-year	1-year	3-year	5-year	Recurrence rate
Soin 2020	33	75.0	53.0	53.0	78.0	78.0	52.0	24.0%
Bhatti 2022	28	–	–	–	–	–	–	18.5%
Lei 2012	72	90.8	75.1	70.5	–	–	–	15.9%
Han 2016	17	–	–	–	87.5	–	–	37.5%
Jeong 2017	24.5	87.4	60.5	–	70.6	57.8	–	35.3%
Inomata 2021	62.5	–	–	–	–	–	–	0.0%
Na 2016	37	83.3	75.0	75.0	81.8	81.8	81.8	–
Llovet 2018	81	95.5	86.4	80.2	–	–	–	31.8%
Kulkarni 2024	10	–	–	–	–	–	–	0.0%
Lee S 2020	89	94.4	84.0	83.3	88.1	85.2	83.5	–
Cho 2018	44	–	–	–	–	–	–	51.4%
Ogawa 2019	90	–	100.0	100.0	–	–	86.0	–
Jang 2010	29.3	89.2	–	54.6	74.7	–	66.3	29.7%
Wu 2020	NR	82.1	74.3	56.2	92.3	64.6	54.7	–
Yu 2012	29	94.1	–	92.7	93.9	–	90.1	7.8%
Lim 2024	NR	100.0	90.9	83.6	–	–	–	–
Cheng 2025	15.5	75.0	–	–	–	–	–	20.0%

Values represent the downstaged subgroup. FU, follow-up; OS, overall survival; RFS, recurrence-free survival; NR, not reported; – = not reported. Follow-up is reported in months and is the median unless otherwise noted; values for Soin 2020 and Inomata 2021 are means. The Cho 2018 follow-up is for the entire LDLT cohort, as no separate value was reported for the beyond-Milan subset. Recurrence rate = cumulative rate over the entire follow-up period.

### Downstaging success rate

The pooled downstaging success rate was 47.5% (95% CI: 36.1%–59.1%; 12 studies, 1,186 patients attempted; I^2^ = 90.7%, p < 0.001) (Figure 5), ranging from 10.4% for ICI-based protocols (Kulkarni et al.) to 73.9% for DEB-TACE (Yu et al., 2016). The considerable heterogeneity reflects differences in initial tumor burden, downstaging modality, and the definition of “success” across studies.

**FIGURE 5:**
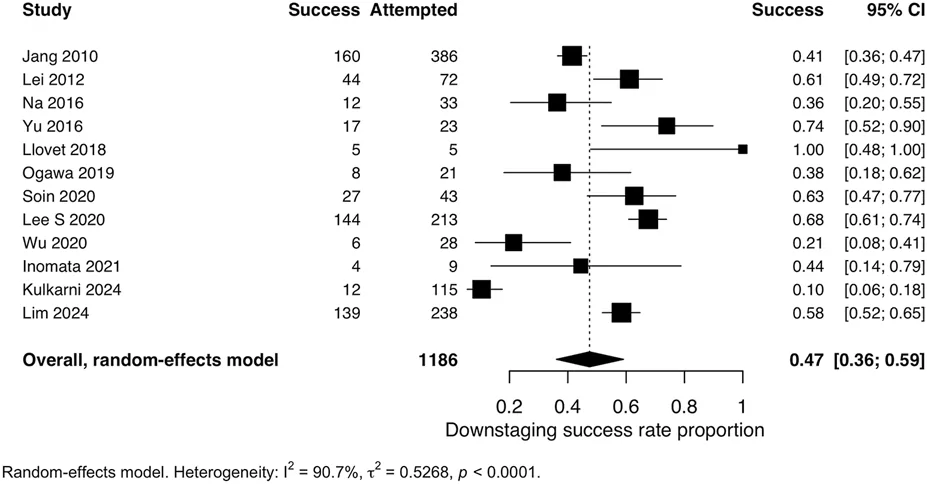
Forest plot of pooled downstaging success rate. Considerable heterogeneity (I^2^ = 90.7%) reflects differences in initial tumor burden, downstaging modality, and success definitions across studies.

### Secondary and exploratory analyses

In the three PVTT-focused studies using radiation-based protocols (Soin 2020, Han 2016, Jeong 2017; N = 50), the pooled recurrence rate was 30.4% (95% CI: 19.1%–44.5%). The two ICI-based studies (Kulkarni 2024, Cheng 2025; N = 14) reported 0/4 and 2/10 recurrences with short median follow-up (10 and 15.5 months), so they were not pooled. Pooled recurrence in PVTT-present (6 studies) and PVTT-absent cohorts (6 studies) was 25.3% and 24.0% (p = 0.88); power was limited because only successful responders reach transplant in either group (Supplementary Figure S1).

### Criterion-based subgroup analysis

Among the 12 studies with recurrence data, those using expanded biology-based or institution-specific criteria showed 28% recurrence (95% CI: 20%–38%) versus 21% (95% CI: 11%–38%) for studies targeting Milan or UCSF criteria (p = 0.44). Target criteria alone did not separate recurrence risk, although too few studies fell into each subgroup for a firm conclusion (Supplementary Figure S2).

### Sensitivity analysis and publication bias

Leave-one-out sensitivity analysis for the pooled 5-year OS showed no single influential study (pooled values 71.8%–77.5% on sequential omission; Supplementary Figure S3). Funnel plots for recurrence (Supplementary Figure S4) and 5-year OS (Supplementary Figure S5) were symmetric, and Egger’s test was not significant (p = 0.17), although power to detect publication bias was limited by the number of studies.

### Reconstructed individual patient data analysis

Patient-level survival was reconstructed from the Kaplan–Meier curves of 10 studies (328 patients) for overall survival and 4 studies (226 patients) for recurrence-free survival. In the one-stage pooled analysis, which accounts for censoring and uneven follow-up, overall survival was 91.6%, 79.7%, and 75.7% (95% CI: 70.5%–80.9%) at 1, 3, and 5 years, and recurrence-free survival 85.6%, 78.1%, and 75.6% (95% CI at 5 years: 69.3%–81.8%) (Figure 6). The median was not reached. These time-to-event estimates closely matched the proportion-based pooled estimates (5-year overall survival 75.0%; recurrence-free survival 70.8%).

**FIGURE 6:**
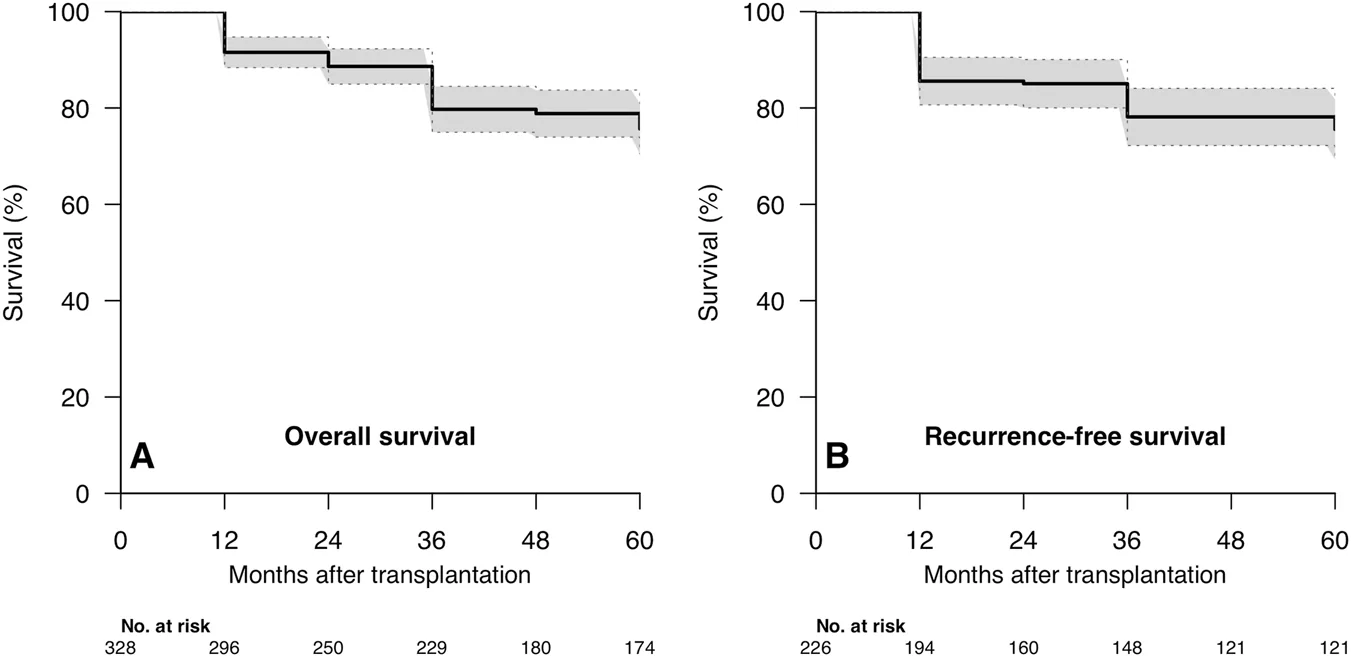
Pooled reconstructed individual-patient-data Kaplan–Meier curves for **(A)** overall survival and **(B)** recurrence-free survival after locoregional downstaging and LDLT. Curves were reconstructed from published plots by the method of Guyot et al. and pooled in a one-stage analysis; shaded areas denote 95% confidence intervals, and numbers at risk are shown below each panel.

## Discussion

This review asks a narrow question: what happens after transplant when a patient beyond Milan criteria responds to locoregional therapy and then receives a living donor graft? Three features make it worth asking. First, a healthy donor accepts an operation for one named recipient, and no allocation rule constrains listing, so each center must judge the expected oncological return for itself. Second, response-based selection may work differently when centers are free to accept any responder; whether the signal that justifies downstaging survives that freedom is the central question. Third, recurrence varied less between studies (I^2^ = 55.7%) than 5-year OS (I^2^ = 77.2%) or 5-year RFS (I^2^ = 74.7%). We therefore treat recurrence as the most comparable signal, not a stable one—in the smaller primary cohort it varied more (22.1%; I^2^ = 81.4%)—and the pooled survival estimates need more caution. We analyzed 511 downstaged LDLT recipients in 17 studies, nine meeting the primary definition.

These numbers describe patients who had already succeeded. Downstaging is both a treatment and a filter: patients who progress, respond poorly, or decompensate are usually not transplanted, so they never enter the studies we pooled. Across the 1,186 patients in the 12 studies that reported how many started downstaging, only 47.5% succeeded. The good outcomes we report therefore reflect, at least in part, the selection of tumors with favorable biology. They are not proof that downstaging itself produced the benefit.

It matters which question a pooled number answers. What happens to everyone who starts downstaging requires an intention-to-treat design that counts the dropouts; our data cannot answer that. They answer a second question—what happens to a patient who has already responded and reached transplant—which is the answer a team needs when a responding beyond-Milan candidate has a living donor. The numbers should not be read as evidence that downstaging beats no downstaging. Twelve of the 17 studies reported how many patients started, but none reported what happened to those who failed, and none included beyond-Milan patients transplanted without successful downstaging. Future studies should enroll patients when downstaging begins and report every dropout with its cause and outcome.

Waiting time changes what a good response means, and LDLT has almost none. UNOS/OPTN policy requires at least 6 months of observation after successful downstaging—the “ablate-and-wait” principle—during which aggressive tumors declare themselves and leave the list. A deceased donor candidate therefore passes two tests, response to treatment and stability over time. LDLT removes the second: donor availability rather than a queue sets the timing, and transplant may follow within weeks. Response to downstaging then becomes the main, and often the only, window onto tumor biology. Two things follow. First, how a tumor responds matters more than the bare fact of response. Durability across serial scans, the AFP trend, and des-gamma-carboxy prothrombin (PIVKA-II) all carry information; a recent single-center series showed that both markers predict long-term outcome after LDLT for HCC [20]. Our own data fit this reading: neither the target criteria (p = 0.44) nor the downstaging modality (p = 0.55) separated recurrence risk. Second, a short deliberate wait may help when the response is recent, marginal, or achieved only after several treatments, because it would restore part of the biological filter that LDLT removes. This has not been tested and deserves prospective study, particularly in the Asian LDLT-predominant programs that produced most of our cohorts.

Our survival estimates sit close to the deceased donor downstaging literature. Yao et al. reported 5-year post-transplant survival of 77.8% in the UCSF experience [7]. Mehta et al. reported 3-year survival of 79% in the UNOS-DS group and 71% in the “all-comers” group, against 83% for patients always within Milan criteria [9]. Parikh et al. pooled 24 mostly DDLT studies and reported 5-year survival of 60%–80% [10], and Tan et al. reviewed 25 studies using the UNOS downstaging criteria [11]. Our pooled 5-year OS of 75.0% falls inside that range, whereas our recurrence rate of 25.0% sits above the 16%–20% those series report [7, 10]. These cohorts differ in selection, protocol, and follow-up, and we made no direct comparison; the data show broad similarity, not equivalence, between donor types. The overlap in survival fits the view that tumor biology and treatment response drive outcome more than graft type does [21].

Heterogeneity is the main objection to any downstaging meta-analysis, and we designed our subgroup analyses around it. By modality, recurrence was 30% for radiotherapy combined with LRT, 23% for LRT alone, and 17% for ICI-based regimens (p = 0.55); across very different treatments, outcome appears to be determined by the selection of tumors that respond rather than by the technique used [22, 23]. By target criteria, biology-based or expanded criteria showed 28% recurrence against 21% for Milan or UCSF (p = 0.44). Sensitivity analyses agreed with the main result: excluding studies under 10 patients gave recurrence 25.1%, 5-year OS 74.0%, and 5-year RFS 69.0%; excluding studies under 20 patients gave recurrence 24.2%; and excluding ICI-based protocols changed nothing (Supplementary Table S3). The pre-specified restricted analysis gave a recurrence rate of 22.1%, close to the overall 25.0%, so including higher-risk study types did not inflate the pooled estimate; heterogeneity for survival nevertheless stayed high even in that cohort, so survival appears to track center-level differences in patient selection more than modality or donor type. We did not pool by individual technique—chemoembolization, radioembolization, ablation, or radiotherapy—because in 11 of the 17 studies patients received combined or sequential treatments and outcomes were not reported by technique; labeling each cohort by a “main” modality would have required an arbitrary rule. Supplementary Table S4 lists the full treatment composition of every cohort.

These findings matter most where LDLT is the main route to transplant, as in South Korea, Japan, Taiwan, and India [13, 14]. DDLT has a standardized UNOS/OPTN downstaging framework; LDLT has none, and our results support an LDLT-specific consensus that uses biological markers alongside imaging criteria. ICI-based downstaging needs particular attention. The two included studies reported pathological response rates of 40%–50%, but one reported allograft rejection in half the recipients [24] and the other wound healing problems without rejection [25]; washout, immunosuppression, and long-term rejection risk remain unknown. AFP is built into several expanded LDLT criteria, but too few included studies reported it systematically for us to test its value as a selection marker.

Three features of the evidence limit how far these results travel. First, sixteen of the 17 cohorts were Asian, one was European, and none came from the Americas. LDLT practice, hepatitis etiology, tumor biology, and organ access differ, so Western programs should apply our estimates with caution. Second, no included study reported outcomes by graft type or operative detail—graft side and size, graft-to-recipient weight ratio, or extent of hilar dissection—so our finding that modality did not determine outcome (p = 0.55) says nothing about surgical factors. Third, the field needs standardization more than another single-center series: uniform mRECIST response assessment, an agreed definition of downstaging success, mandatory reporting of the intention-to-treat denominator and of dropout, routine explant pathology, and fixed-interval oncological endpoints.

This study has several limitations. First, and most important, every outcome is conditional on successful downstaging and transplant. No study reported what happened to patients who failed, progressed, or dropped out, so our estimates describe responders who reached transplant, not an intention-to-treat population, and both selection bias and immortal time bias apply. Second, every included study was retrospective and single-center. Third, downstaging criteria, modalities, and definitions of success varied widely; subgroup and sensitivity analyses suggest this did not meaningfully move the recurrence estimate, but heterogeneity for pooled survival stayed high, and individual locoregional techniques could not be compared because most cohorts received combined or sequential treatment (Supplementary Table S4). Fourth, the studies were small, with a median of 25 patients, although excluding studies under 20 and under 10 patients gave consistent results (Supplementary Table S3). Fifth, our main survival syntheses pooled proportions at fixed time points, which ignores censoring; reconstructed patient-level data gave closely matching estimates (5-year OS 75.7%, RFS 75.6%), but reconstruction was possible only where usable curves existed and relied on time-point rates where risk tables were missing. Sixth, explant pathology (microvascular invasion, differentiation, necrosis), AFP, and DCP were reported inconsistently, so radiologic response could not be linked to true tumor burden; systematic explant reporting would most directly validate downstaging. Seventh, one investigator performed selection and extraction, with pilot calibration and cross-checking. Finally, publication bias cannot be excluded, although Egger’s test was not significant.

## Conclusion

After locoregional downstaging before LDLT for HCC beyond Milan criteria, pooled 5-year OS was 75.8% and recurrence 22.1% in the pre-specified primary analysis, and across all 17 studies recurrence (25.0%) varied least between studies, making it the most comparable oncological signal available. Successful downstaging is a filter as well as a treatment—fewer than half of the patients who attempted it succeeded—and LDLT removes the waiting interval that filters tumors a second time in the deceased donor setting. Selection should therefore rest on how durable and how biologically convincing the response is, rather than on meeting a size-and-number rule alone. Prospective multicenter intention-to-treat studies with standardized protocols and endpoints are needed before routine clinical application.

## Data Availability

The original contributions presented in the study are included in the article/Supplementary Material, further inquiries can be directed to the corresponding author.
