# Supplementary material for: Post-transplant oncologic outcomes after locoregional downstaging before living donor liver transplantation for hepatocellular carcinoma beyond Milan criteria: a systematic review and meta-analysis of selected cohorts
Source: Transpl Int. 2026 Oct 20;39:17284. doi: 10.3389/ti.2026.17284 (PMC13727510; doi:10.3389/ti.2026.17284)
Supplement: Supplementary file 1 [file DataSheet1.pdf]

SUPPLEMENTARY MATERIALS

Post-Transplant Oncologic Outcomes After Locoregional Downstaging Before Living Donor Liver Transplantation for Hepatocellular Carcinoma Beyond Milan Criteria: A Systematic Review and Meta-Analysis of Selected Cohorts

Capsule Sentence Summary

Among 511 recipients transplanted after successful downstaging, pooled recurrence was 25% and five-year survival 76%. Fewer than half of the patients who attempted downstaging completed it successfully, so treatment response acts as a biological filter.

Supplementary Table S1. Search Strategy

PubMed Search (performed April 8, 2026)

("living donor liver transplantation" OR "living donor" OR "LDLT" OR "living related liver transplantation") AND ("hepatocellular carcinoma" OR "HCC" OR "liver cancer" OR "liver neoplasms") AND ("downstaging" OR "down-staging" OR "locoregional therapy" OR "bridging therapy" OR "transarterial chemoembolization" OR "TACE" OR "radiofrequency ablation" OR "RFA" OR "radioembolization" OR "SBRT" OR "neoadjuvant")

Embase Search (performed April 8, 2026)

TITLE-ABS-KEY(("living donor liver transplantation" OR "living donor" OR "LDLT") AND ("hepatocellular carcinoma" OR "HCC" OR "liver cancer") AND ("downstaging" OR "down-staging" OR "locoregional" OR "bridging" OR "TACE" OR "RFA" OR "neoadjuvant"))

Supplementary Table S2. Study Contributions to Pooled Analyses

Each study contributed to pooled analyses only for outcomes for which extractable data were available in the original publication.

Study	1yr OS	3yr OS	5yr OS	5yr RFS	Recurrence	DS Success
Soin 2020	+	+	+	+	+	+
Bhatti 2022	–	–	–	–	+	–
Lei 2012	+	+	+	–	+	+
Han 2016	–	–	–	–	+	–
Jeong 2017	+	+	–	–	+	–
Inomata 2021	–	–	–	–	+	+
Na 2016	–	–	–	+	–	+
Llovet 2018	+	+	+	–	+	+
Kulkarni 2024	–	–	–	–	+	+
Lee S 2020	+	+	+	+	–	+
Cho 2018	–	–	–	–	+	–
Ogawa 2019	–	+	+	+	–	+
Jang 2010	+	–	+	+	+	+
Wu 2020	–	–	+	+	–	+
Yu 2012	+	–	+	–	+	+
Lim 2024	+	+	+	–	–	+
Cheng 2025	+	–	–	–	+	–

+ = study contributed to this analysis; – = data not available or not extractable. DS = downstaging; OS = overall survival; RFS = recurrence-free survival.

Supplementary Table S3. Sensitivity analyses excluding small studies.

Outcome	Analysis	Studies (k)	Pooled estimate (95% CI)	I²
Recurrence rate	All studies	12	25.0% (17.4–34.5)	55.7%
Recurrence rate	Excluding <10 patients	9	25.1% (16.7–35.7)	65.3%
Recurrence rate	Excluding <20 patients	7	24.2% (14.8–36.9)	73.0%
5-year overall survival	All studies	9	75.0% (63.1–84.1)	77.2%
5-year overall survival	Excluding <10 patients	8	74.0% (61.6–83.4)	79.2%
5-year overall survival	Excluding <20 patients	8	74.0% (61.6–83.4)	79.2%
5-year recurrence-free survival	All studies	6	70.8% (55.1–82.7)	74.7%
5-year recurrence-free survival	Excluding <10 patients	5	69.0% (52.0–82.0)	79.0%
5-year recurrence-free survival	Excluding <20 patients	4	66.4% (47.1–81.5)	83.6%

Random-effects meta-analysis of proportions (logit transformation, DerSimonian–Laird estimator). Studies excluded at the <10-patient threshold: Han 2016, Inomata 2021, and Kulkarni 2024 (recurrence); Ogawa 2019 (5-year overall survival and recurrence-free survival). CI, confidence interval.

Supplementary Table S4. Downstaging modality composition of each included study.

Study	Country	Downstaging modality	Regimen pattern	Target criteria	n downstaged
Soin 2020	India	SBRT/TARE/TACE/RFA	Combined or sequential	PVTT response	25
Bhatti 2022	Pakistan	TACE/RFA/MWA	Combined or sequential	UCSF-expanded	27
Lei 2012	China	TACE/resection/RFA/EI	Combined or sequential	Milan / UCSF	44
Han 2016	Korea	CCRT/HAIC	Combined	PVTT response	8
Jeong 2017	Korea	3D-CRT/TACE	Combined	Vascular invasion response	17
Inomata 2021	Japan	TACE/RFA/MCT	Combined or sequential	Milan	4
Na 2016	Korea	TACE/RFA/resection/PEI	Combined or sequential	Milan	12
Llovet 2018	Spain	TACE/PEI	Combined	BCLC expanded	22
Kulkarni 2024	India	Atezo-Bev	Single agent (systemic/ICI)	Milan after ICI	4

Study	Country	Downstaging modality	Regimen pattern	Target criteria	n downstaged
Lee S 2020	Korea	cTACE/RFA/combination	Combined or sequential	Milan	144
Cho 2018	Korea	TACE	Single modality	Milan	37
Ogawa 2019	Japan	TACE/TAE/RFA/PEI/resection	Combined or sequential	Kyoto (5-5-500)	8
Jang 2010	Korea	TACL	Single modality	Milan / complete necrosis	37
Wu 2020	Taiwan	TACE/ablation	Combined or sequential	Milan	28
Yu 2012	Taiwan	LRT (mixed)	Not disaggregated	Milan	51
Lim 2024	Taiwan	DEB-TACE	Single modality	Milan	33
Cheng 2025	Taiwan	ICI	Single agent (systemic/ICI)	Milan	10

Modality designations reproduce Table 1 of the main text. In 11 of the 17 included studies patients received combined or sequential modalities and outcomes were not reported separately by technique; 5 studies employed a single modality or agent, and 1 study reported locoregional therapy without disaggregation. Technique-level pooling was therefore not attempted, and modality-based synthesis was restricted to the modality-class subgroups reported in the main text (locoregional therapy alone, radiotherapy combined with locoregional therapy, and immune checkpoint inhibitor-based regimens). 3D-CRT, three-dimensional conformal radiotherapy; Atezo-Bev, atezolizumab-bevacizumab; BCLC, Barcelona Clinic Liver Cancer; CCRT, concurrent chemoradiotherapy; cTACE, conventional TACE; DEB-TACE, drug-eluting bead TACE; EI, ethanol injection; HAIC, hepatic arterial infusion chemotherapy; ICI, immune checkpoint inhibitor; LRT, locoregional therapy; MCT, microwave coagulation therapy; MWA, microwave ablation; PEI, percutaneous ethanol injection; PVTT, portal vein tumor thrombosis; RFA, radiofrequency ablation; SBRT, stereotactic body radiotherapy; TACE, transarterial chemoembolization; TACL, transarterial chemolipiodolization; TAE, transarterial embolization; TARE, transarterial radioembolization; UCSF, University of California San Francisco.

Supplementary Figures

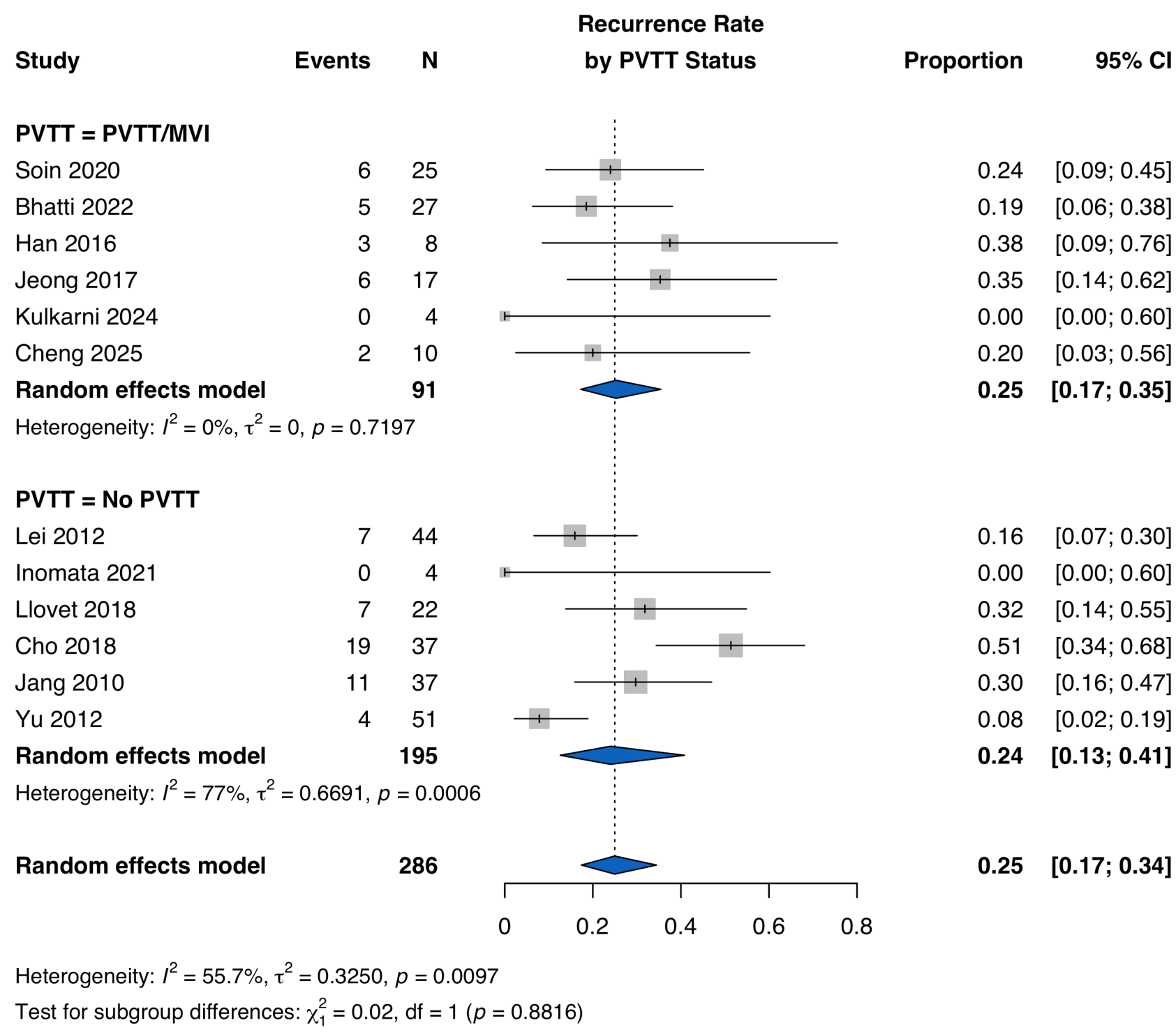

Supplementary Figure S1. Subgroup analysis of recurrence rate by portal vein tumor thrombosis (PVTT) status. Pooled recurrence was 25.3% in cohorts with PVTT (6 studies) and 24.0% in cohorts without PVTT (6 studies); the subgroup difference was not significant (p = 0.88). Because only patients who respond to downstaging reach transplantation in either group, power to detect a difference is limited.

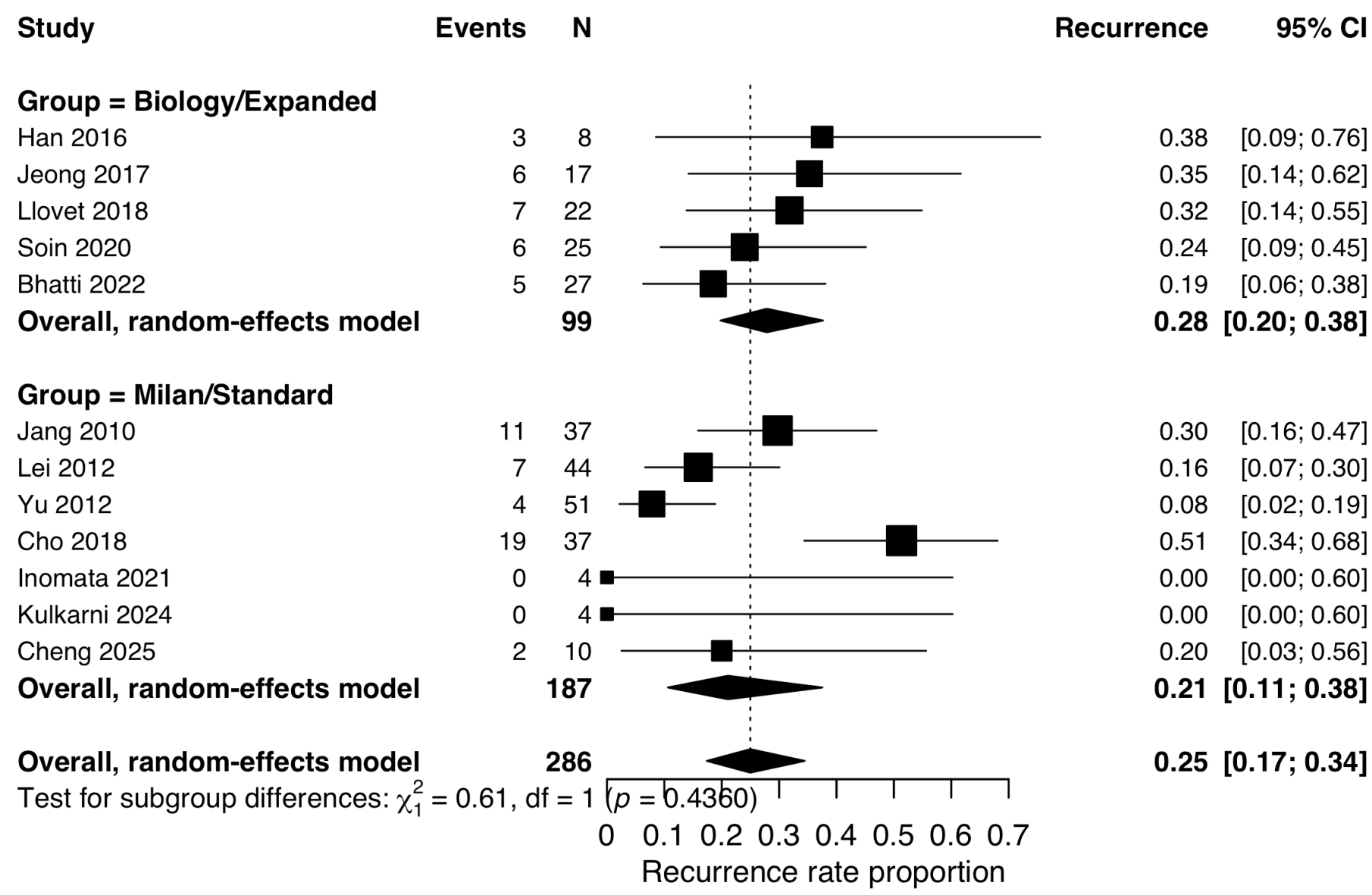

Random-effects model. Heterogeneity: I^2 = 55.7%, τ^2 = 0.3250, p = 0.0097.

Supplementary Figure S2. Subgroup analysis of recurrence rate by target transplant criteria (Milan/UCSF standard radiologic criteria vs. expanded biology-based or institution-specific criteria) across the 12 studies with reported recurrence data. Pooled recurrence was 21% (95% CI: 11–38%) and 28% (95% CI: 20–38%), respectively; subgroup difference test p = 0.44.

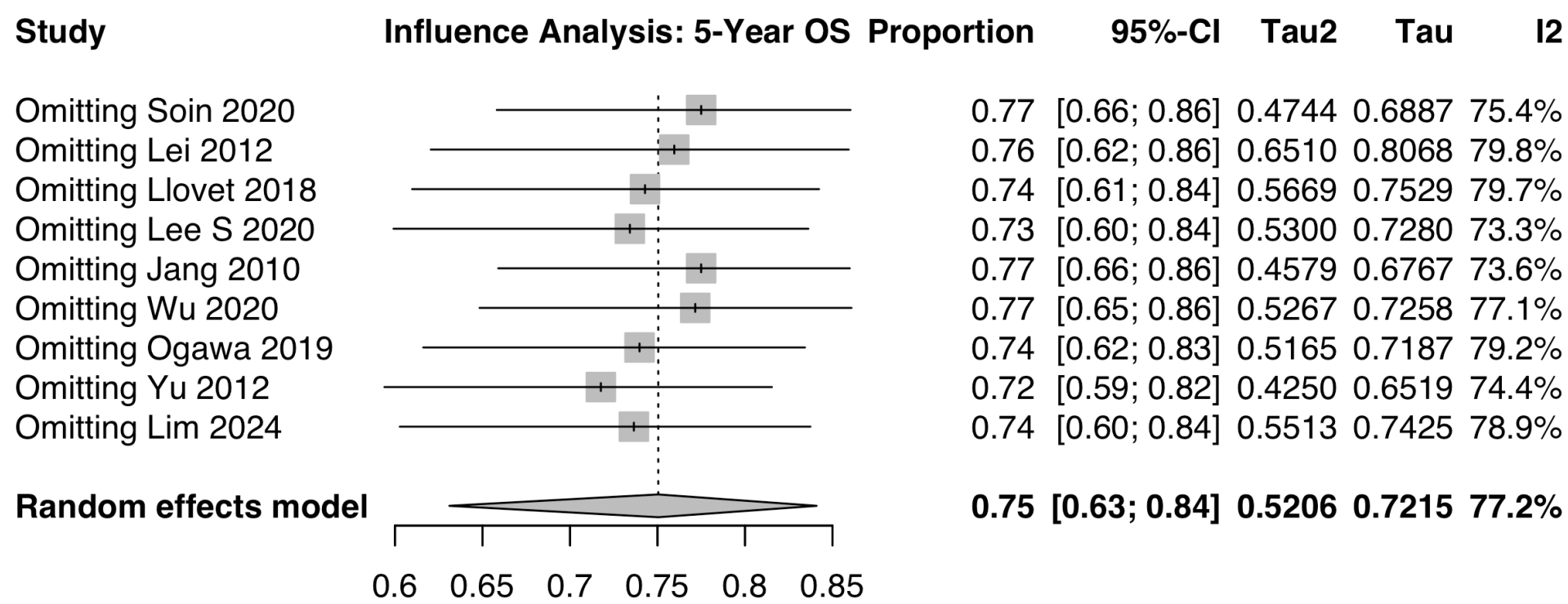

Supplementary Figure S3. Leave-one-out sensitivity analysis for pooled 5-year overall survival. Pooled estimates ranged from 71.8% to 77.5% on sequential omission of individual studies, indicating that no single study disproportionately influenced the pooled result.

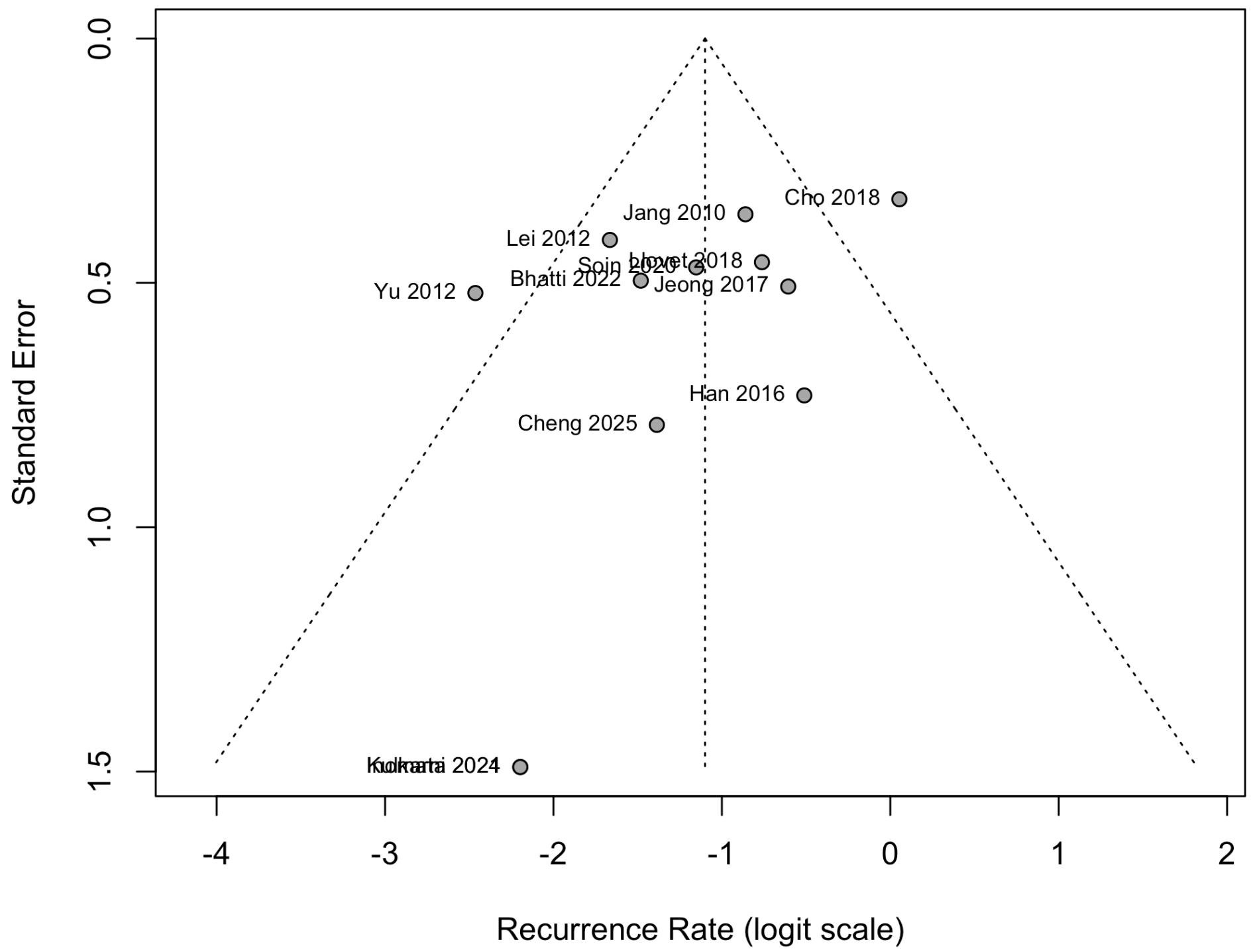

Supplementary Figure S4. Funnel plot for the pooled recurrence rate. The plot appeared symmetric and Egger's test showed no significant funnel plot asymmetry (p = 0.17), although power to detect publication bias was limited by the number of included studies.

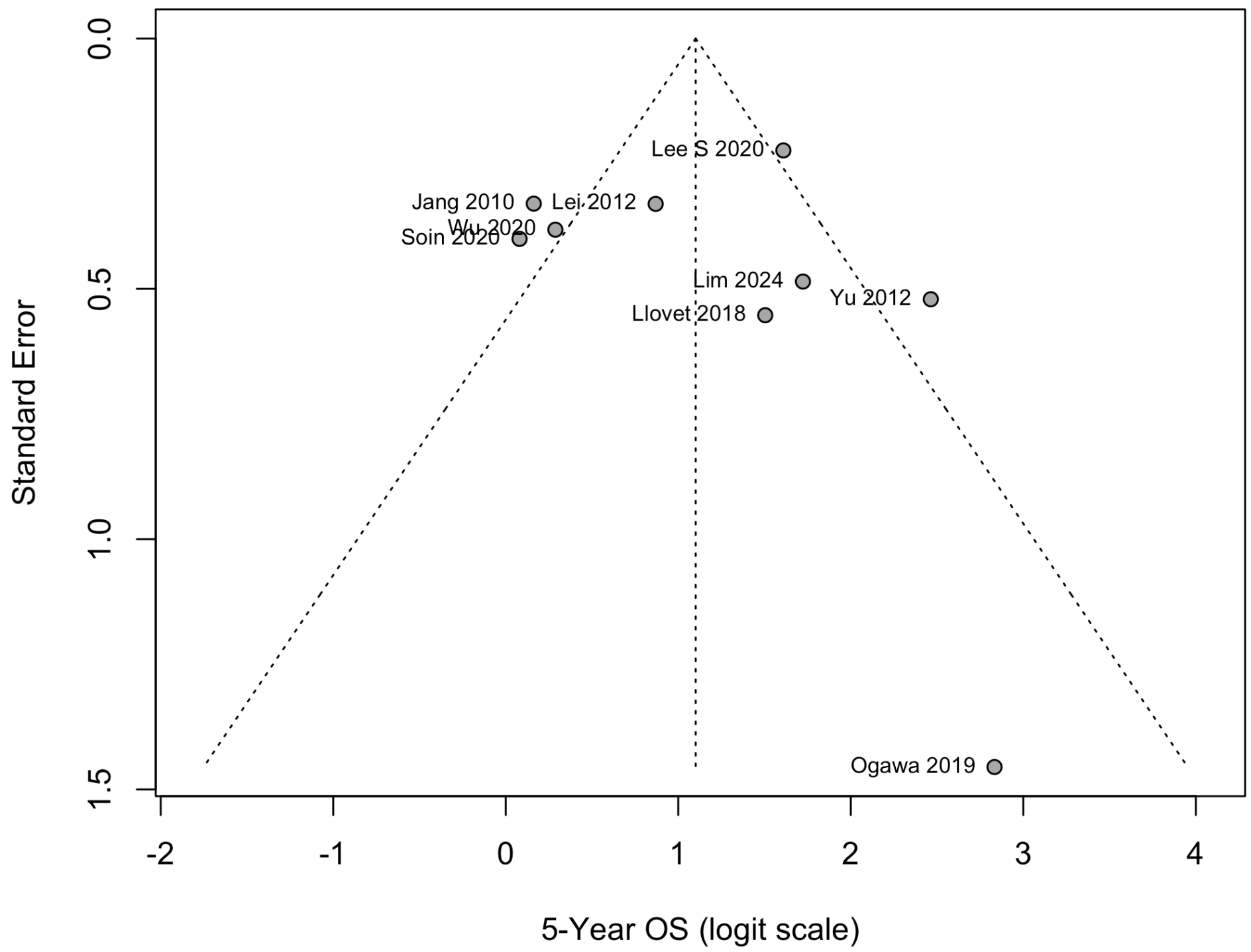

Supplementary Figure S5. Funnel plot for pooled 5-year overall survival. No notable asymmetry was apparent on visual inspection; as above, power to detect publication bias was limited.
